# Identification of Key Functional Motifs of Native Amelogenin Protein for Dental Enamel Remineralisation

**DOI:** 10.3390/molecules25184214

**Published:** 2020-09-14

**Authors:** Shama S. M. Dissanayake, Manikandan Ekambaram, Kai Chun Li, Paul W. R. Harris, Margaret A. Brimble

**Affiliations:** 1 School of Chemical Sciences, 23 Symonds St, The University of Auckland, Auckland 1142, New Zealand; sdis008@aucklanduni.ac.nz; 2 Paediatric Dentistry, Biomaterials, Faculty of Dentistry, The University of Otago, Dunedin 9016, New Zealand; mani.ekambaram@otago.ac.nz (M.E.); kc.li@otago.ac.nz (K.C.L.); 3 School of Biological Sciences, 3b Symonds St, The University of Auckland, Auckland 1142, New Zealand; 4 Maurice Wilkins Centre for Molecular Biodiscovery, 3b Symonds St, The University of Auckland, Auckland 1142, New Zealand

**Keywords:** dental caries, enamel remineralisation, hydroxyapatite, amelogenin, amelogenin-derived peptides, leucine-rich amelogenin peptides, tyrosine-rich amelogenin peptides

## Abstract

Dental caries or tooth decay is a preventable and multifactorial disease that affects billions of people globally and is a particular concern in younger populations. This decay arises from acid demineralisation of tooth enamel resulting in mineral loss from the subsurface. The remineralisation of early enamel carious lesions could prevent the cavitation of teeth. The enamel protein amelogenin constitutes 90% of the total enamel matrix protein in teeth and plays a key role in the biomineralisation of tooth enamel. The physiological importance of amelogenin has led to the investigation of the possible development of amelogenin-derived biomimetics against dental caries. We herein review the literature on amelogenin, its primary and secondary structure, comparison to related species, and its’ in vivo processing to bioactive peptide fragments. The key structural motifs of amelogenin that enable enamel remineralisation are discussed. The presence of several motifs in the amelogenin structure (such as polyproline, N- and C-terminal domains and C-terminal orientation) were shown to play a critical role in the formation of particle shape during remineralization. Understanding the function/structure relationships of amelogenin can aid in the rational design of synthetic polypeptides for biomineralisation, halting enamel loss and leading to improved therapies for tooth decay.

## 1. Introduction

Dental caries has a high prevalence of affecting permanent teeth of an estimated 2.3 billion people and primary teeth of more than 530 million children worldwide [1]. It is the result of manifestation of dental plaque on the tooth enamel. Plaque is a form of biofilm created by bacteria such as *Streptococcus mutans*, *Streptococcus sobrinus* [2] and *Lactobacilli* [3]. These cariogenic bacteria produce acid (formic-, acetic- and propionic acid) as a by-product during the metabolic process of fermentable carbohydrates, which leads to incipient caries or white spot lesions on tooth enamel [4], resulting in damaging the enamel, leading to formation of nanoscale pores [5].

**Figure 1:**
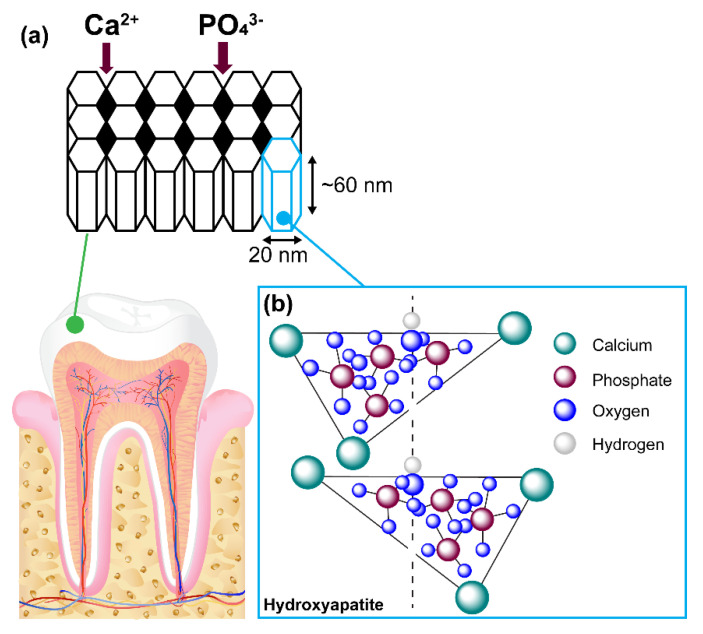
Schematic representation of enamel microstructure. (**a**) Arrangement of the hydroxyapatite crystals, the cavities shown by the black diamond shape, allows Ca^2+^ and PO_4_^3−^ ions from saliva to pass through and assist in remineralization. (**b**) Molecular arrangement of individual unit cells making up the hydroxyapatite.

Tooth enamel is highly mineralised and recognised as an acellular tooth tissue and is not capable of self-regeneration when damaged by injury or decay [6]. Ameloblasts secrete a group of unique enamel matrix proteins (EMPs) that consist of 90% of a protein called amelogenin, and 10% non-amelogenin proteins known as enamelins, ameloblastin and amelotin [7,8]. EMPs regulate the intricate matrix-like formation of hydroxyapatite (HA) crystals contributing to 70–80% of enamel weight within the human tooth. HA is a calcium phosphate mineral with the following chemical composition:

Ca_10_(PO_4_)_6_(OH)_2_ and a P/Ca molar ratio of 1:1.67 [9]. Each single cell unit is observed to be long, hexagonal, nanorod-like structures consisting of repeating units of morphological dimensions of ~ 60 nm in length, 20 nm in diameter and 2–5 nm thick. The collation of these single cell units forms bundles of prisms or rods that are 60–100 nm long [10]. Each rod is surrounded by a sheath formed from the non-amelogenin enamelin proteins, giving its characteristic matrix appearance (Figure 1).

In between these rods, nanoscale pores are formed that enable the permeability of extracellular fluid containing important chemicals such as hydroxyl ions, chloride, carbonate and fluoride ions. Proteins such as statherin in saliva hold calcium and phosphate ions in a supersaturated form, making them readily available in a constant and sufficient supply to help the regeneration of HA [11]. Saliva also contains acidic and basic proline-rich proteins (PRPs), which provides a protective rind by attaching itself onto oral bacteria. The strength and rigidity of HA is mainly due to intricately woven crystals consisting of calcium, phosphate and oxygen.

### 1.1. Process of Microbial Attack on Dental Enamel

During the microbial invasion of the enamel, the nanoscale pores allow bacteria and acidic fluid to freely circulate through the channels within the matrix. Once the acid has reached a susceptible site, calcium and phosphate ions are dissolved into the surrounding extracellular environment from the hydroxyapatite (HA) crystal matrix, leading to the loss of calcium and phosphate ions, thus hindering the natural mineralisation process of enamel. This phenomenon is known as demineralisation, where the HA crystalline structure will decrease in size while the pores enlarge [12]. This combination of food fermentation and attack by cariogenic bacteria initiates the early manifestation of dental caries by beginning to demineralise the dental enamel [13].

### 1.2. Potential Approaches for Dental Remineralisation

Due to high prevalence of dental caries amongst any given population globally, the development of a potential therapeutic for dental caries is essential. Therefore, the use of organic scaffolds [14], dendrimers [15], chitosan [16], charged amino acids [17] and bioactive glasses [18] have been explored for remineralisation properties. Unfortunately, these approaches are yet to yield any commercial products. However, biomimetic in vitro strategies employing proteins which contribute to the EMP layer such as native amelogenin and leucine-rich amelogenin peptide (LRAP) have demonstrated the ability to bind hydroxyapatite crystals, which is crucial for remineralisation. The use of LRAP in vitro has demonstrated the de novo formation of HA [19]. This is of significant importance to researchers and dental clinicians to develop biomimetic peptides as potential treatments to initiate remineralisation. The critical role amelogenin protein plays in controlling enamel remineralisation has become evident over the last four decades [20].

## 2. Determination of Primary and Secondary Amelogenin Protein Structures amongst Different Species

**Figure 2:**
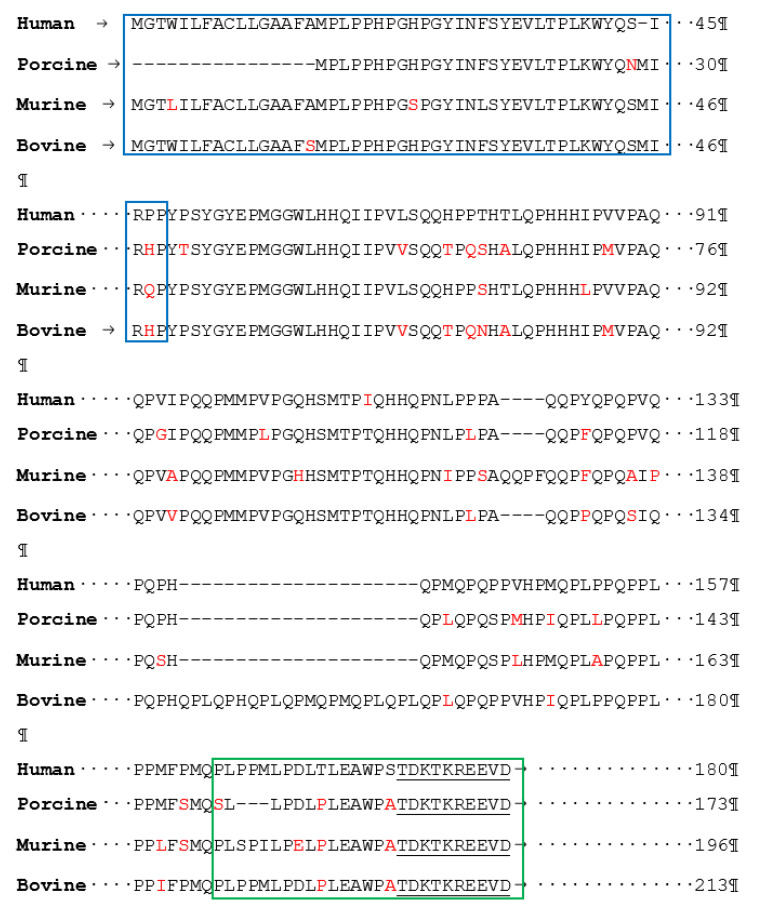
Alignment of native amelogenin protein from different species. The blue boxed areas indicate the N-terminal domain, the green boxed area indicates the C-terminal domain, underlined residues indicate the highly charged C-terminal tail, and red indicates the amino acid residues, which are non-homologous in comparison to the native human amelogenin protein. The (-) dash indicates the alignment gap.

Amelogenin protein first gained recognition in 1980 for its vital role in dental mineralisation during enamel matrix development [21]. These proteins self-assemble into ~17–18 nm globular supramolecular aggregates (nanospheres), microribbons and nanochains during enamel formation [22]. This self-assembling capability of amelogenin is known to be a key driving force in guiding the formation of HA crystals [23]. Therefore, dental clinicians and researchers have taken an interest in the ability of these proteins to effect enamel remineralisation. In the past four decades, several studies have been conducted to characterise the primary and secondary structure of the native amelogenin protein amongst various species to determine its active motifs [24]. Studies carried out to deduce the primary native amelogenin proteins demonstrated that amelogenins vary in sequence length (150–180 amino acids) depending on the origin of different species, human [25], bovine [26], porcine [27] and murine [28] (Figure 2). The primary structure of amelogenin protein is shown to be highly conserved (>80%) amongst different species consisting of three main domains: An N-terminal domain, a mid-section and the C-terminal domains [29]. Determination of the secondary structures would assist in understanding how these individual amelogenin proteins contribute to enamel crystal growth [30]. Techniques such as circular dichroism (CD), nuclear magnetic resonance (NMR) spectroscopy, isothermal titration calorimetry (ITC), selected area electron diffraction (SAED) and Fourier transform-infrared (FT-IR) have been used to fully characterise primary and secondary structure of native amelogenin proteins.

### 2.1. Native Bovine Amelogenin Structure

The primary structure of native bovine amelogenin protein was reported by Takagi et al. [31] in 1984, and is seen to be the largest of the native amelogenins, with a molecular weight of 32 kDa consisting of 213 amino acid residues. This report led to the study of the secondary structure by Renugopalakrishnan et al. [32] with the use of CD, FT-IR and 2D NMR [33] and the use of Raman spectroscopy by Zheng et al. [34].

The N-terminal domain is reported as constituting of β-turns at residues His^6^-Tyr^12^ and Thr^21^-Lys^24^, Gln^40^–Gly^43^, Ile^51^–Val^54^, Thr^58^–Asp^61^, Ile^70^–Val^73^, Gln^77^–Gln^83^, Val^88^–Glu^91^ and Gln^93^–Leu^96^ and polyproline repeat units from Gln^112^ to His^139^ [32].

The presence of certain band values of frequencies between amide I depicting 1645–159 cm^−1^ and amide II depicting 1262–1300 cm^−1^ frequencies in FT-IR indicates an α-helix within the segment Gly^44^-Ile^50^, as deduced by Renugopalakrishnan et al. [32]. Having only seven amino acid residue segments contributing towards the α-helical structure is classed as a minimal contribution to native bovine amelogenin secondary structure.

**Figure 3:**
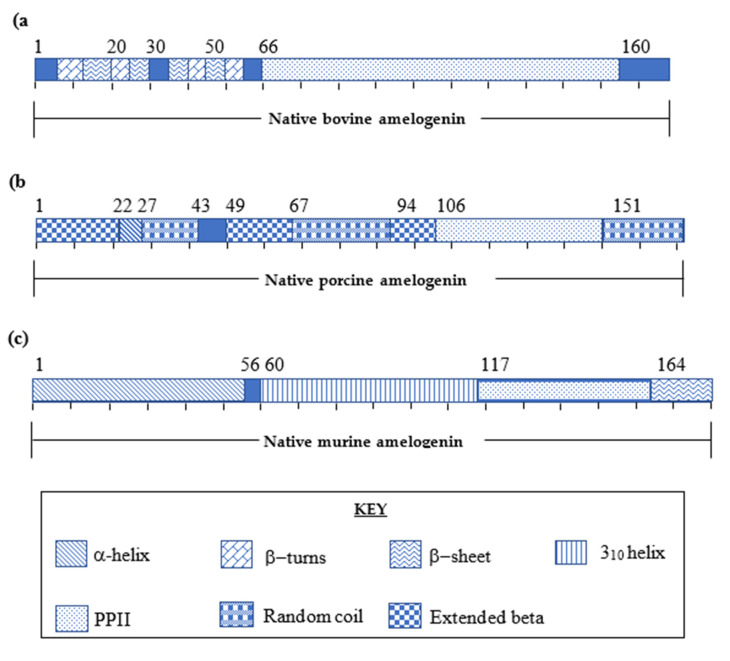
(**a**) Secondary structure of native bovine amelogenin determined by CD, FT-IR and 2D-NMR. (**b**) Secondary structure of native porcine amelogenin determined by VT-CD. (**c**) Secondary structure of native murine amelogenin determined by 3D-NMR.

The mid-section from Pro^66^ to Pro^160^ consists of a very high proportion of proline residues and is reported as forming the poly-l-proline type II (PPII) helical region. A series of β-sheet subdomains coexisting within the β-turns at the C-terminal end have also been described (Figure 3a) [32].

### 2.2. Native Porcine Amelogenin Structure

The study of the primary and secondary structure of native porcine amelogenin was defined by Lakshminarayanan et al. [35] using FT-IR and CD. The native porcine amelogenin was demonstrated to be 173 amino acid residues with a molecular weight of 27kDa. [36] Similarities between both porcine and bovine native amelogenins are indicated by the presence of β-sheets within the N-terminal domain, high proline content and a prominent PPII domain within the midsection [37,38]. Native porcine amelogenin composition is observed to be highly similar to the bovine native amelogenin. The primary structure of porcine is slightly varied by the absence of Met^1^–Ser^16^, as seen in bovine, and a midsection polyproline segment from Gln^139^–Leu^159^ is absent in comparison to bovine native sequence (Figure 2). In addition to secondary structure studies, the self-assembling capabilities were identified as being driven by hydrophobic interactions composed of β-sheets utilising variable temperature circular dichromism (VT-CD) [35] (Figure 3b).

### 2.3. Native Murine Amelogenin Protein

The secondary structure of the 196 amino acid residue native murine protein was determined using 3D-NMR techniques by Zhang et al. [30]. For NMR analysis, the native protein was split into three shorter peptide fragments and synthesised as three separate peptides (N-terminus; Amel-N AA 1–92, the mid-section; Amel-M AA 34-154 and the C-terminus; Amel-C AA 86-193) to facilitate characterisation. These synthetic fragments were mapped against the native murine amelogenin, which revealed four major functional domains contributing to the overall 3-D structure of the protein (Figure 3c) [30].

The N-terminal domain consists of four α-helical motifs (Ser^9^-Val^19^, Thr^21^-Pro^33^, Tyr^39^-Trp^45^, and Val^53^-Gln^56^). The mid-section is composed of both an elongated random coil motif with two 3_10_ helices (Pro^60^-Gln^117^) and a PPII helical region (Pro^118^-Leu^165^). The PPII helices are known to exhibit a well-defined conformation, yet they are flexible, proving advantageous in mineral binding domains [30]. The hydrophobicity of amelogenin is attributed to a high proportion (50–60% of the protein sequence) of proline residues in the mid-section [7,35]. The C-terminal domain (Leu^165^-Asn^193^) contains a charged hydrophilic motif comprised of a 13 residue long peptide sequence rich in glutamic acid, arginine and lysine. The overall structure of the C-terminal domain is recognised to give rise to β-sheets and β-turns [30,39] (Figure 3c).

From these varied techniques used to determine both primary and secondary structures of native amelogenin amongst different species, a range of secondary structures motifs were reported, α-helical, polyproline, 3_10_ helices, β-sheets, and β-spirals [30,33,38,40]. Of particular note is the presence of a highly conserved domain such as the PPII motif in the midsection amongst different species [41]. It is evident that the high proline content in the midsection contributes to these well-defined PPII helical conformations [40].

### 2.4. Contribution of Polyproline Motifs of Amelogenin towards Mineralisation

Intramolecular interactions of native amelogenin were determined using a combination of CD and NMR spectroscopic techniques. It is reported that the mobility and flexibility of the PPII helices in the midsection enable the interaction between α-helices and β-sheets commonly found within the N-terminus [30]. This flexibility enables the formation of elongated fibril like structures, allowing interactions with biological surfaces such as the HA layer. A recent study carried out by Jin et al. [40] showed the important contribution of the polyproline-rich motifs to matrix assembly and their vital role in biological mineralisation over a diverse range of different phyla [42]. Further studies by Lakshminarayanan et al. [20] using isothermal titration calorimetry (ITC) studies have reported that PPII motifs adopt a β-sheet secondary structure to facilitate self-assembly. This transformation is driven by the entropy gained via the hydrophobic amino acid residues, known as the hydrophobic effect which enables a conformation that facilitates mineral binding, resulting in HA crystal growth [43].

To assess these proposed effects of HA crystal growth a series of varying length polyproline repeats based on human amelognein were designed and synthesised by Jin et al. [40]. A polyproline motif unit from the latter part of the mid-section was selected (Pro^12^–Pro^153^) (Table 1). A series of proline repeat motifs—PXX12, PXX24 and PXX33, comprising 12, 24 and 33 amino acid residues, respectively—were prepared whereby proline is represented by P and X is substitutes for any amino acid residue [40]. Analysis of these peptides via atomic force microscopy (AFM) and dynamic light scattering (DLS) methods revealed that increasing the length of the polyproline motifs yielded longer HA crystals. The 12 residue motif PXX12 exhibited shorter HA crystals that were 21.6 ± 6.5 nm in length, whereas the PXX24 exhibited 42.9 ± 8.5 nm long crystalline mineralisation and PXX33 exhibited crystal lengths of 102.1 ± 36.3 nm. It is noteworthy that native full-length murine amelogenin protein shows HA crystal formation with crystals of 102.1 ± 19.3 nm in length, similar in size to PXX33 [40].

The glutamine residues are the second most prevalent amino acid in the PPII helix. Glutamine is recognised as the only other amino acid residue apart from proline to have favourable Chou–Fasman conformation parameters [44]. The Chou–Fasman method is used to predict elements of protein structure by assigning a certain value to each amino acid and then applying an algorithm to the assigned values [45]. From the Chou–Fasman conformational parameters, both glutamine and proline are observed to have higher values assigned for a PPII structure than for any other secondary structure. Glutamine is therefore the preferred amino acid residue together with proline for the preferential formation of PPII helices. To validate this hypothesis, the glutamine residues of PXX33 were substituted with alanine, and the resultant peptide coined PQA (Table 1). Comparison of HA crystal formation by PQA peptide or PXX33 peptide revealed far larger nanosphere structures with PQA peptide. These larger nanosphere structures formed by PQA peptide was seen to give rise to flake-like particles that are 18.6 +/− 3.2 nm in diameter. HA crystals can be formed with flake-like, sphere, needle-like or rod-shaped crystals, highly dependent on the particle size. For a successful formation of HA crystal layer, a certain density of 550 crystallites/μm^2^ is crucial [46]. This is only accomplished by the rod-shaped crystals with a specific particle size (60–100 nm in length) [46]. Thus, a larger particle size, as formed by the PQA peptide, gives rise to flake-like crystals, which are unable to form crystalline mineral and, therefore, do not contribute to HA crystal formation. Thus, this confirms that the presence of Gln in the PX33 sequence is required to ensure correct HA crystal growth.

## 3. Splice Variants of Native Amelogenin from Different Species

Proteolytic degradation of the native amelogenin protein at the enamel maturation stage has been shown to involve two key proteases, matrix metalloproteinase (MMP-20) and kallikrein-related peptidase 4 (KLK-4). These two proteases are responsible for the complete degradation of native amelogenin, to yield two distinctive lower molecular weight amelogenin peptides of 5–6 kDa. Due to the amino acid composition of these peptides, they are designated as tyrosine-rich amelogenin peptides (TRAP) and leucine-rich amelogenin peptides (LRAP) [47].

**Figure 4:**
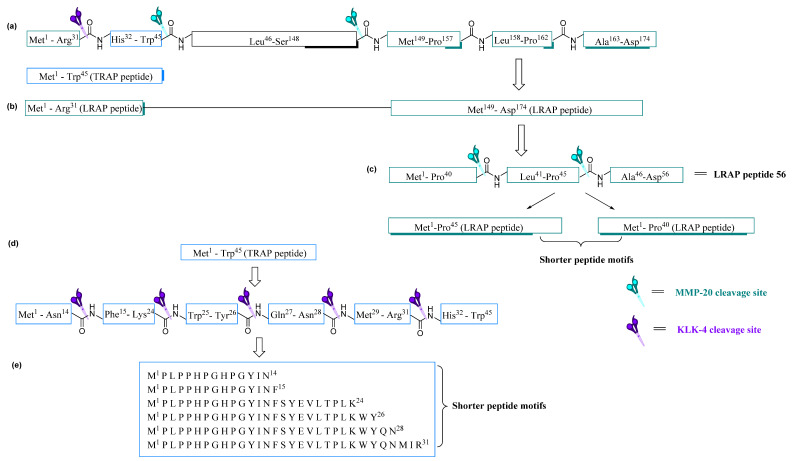
(**a**) Native porcine amelogenin protein indicating its major cleavage sites by KLK-4 and MMP-20 protease generating TRAP and LRAP peptide (**b**) cleaved N-terminal segment and C-terminal segment giving rise to LRAP (**c**) LRAP peptide is further cleaved by MMP-20 to generate shorter motifs of LRAP of 45 and 40 amino acid residues (**d**) TRAP peptide is further cleaved by KLK-4 protease to generate shorter motifs of TRAP peptides (**e**) Amino acid sequence of KLK-4 generated TRAP peptides.

Comparisons of amino acid sequence data for TRAP and LRAP peptides are reported as being conservative amongst bovine, human and porcine for the first 27 amino acid residues (N-terminus), and enrichment of tyrosine and leucine are seen only in the C-terminal regions of these 45 to 46 amino acid residue peptides [48,49]. TRAP peptides are formed by proteolytic cleavage and LRAP formed by proteolytic splicing of the native amelogenin by MMP-20 and KLK-4 (Figure 4a) [50].

The splicing of LRAP is directed by amelogenin promoter for cDNA encoding for LRAP and is beyond the scope of this review [50]. These spliced and degraded TRAP and LRAP peptides undergo further proteolytic cleavage by MMP-20 and KLK-4 to produce shorter fragments [51,52]. The TRAP peptide within the N-terminus of up to 44 to 45 amino acid residues and the LRAP segment span both the N- and C-terminal domains of up to 56 to 59 amino acid residues in length [53].

Yamakoshi et al. [36] has reported that the native 27 kDa porcine amelogenin undergoes splicing to form a major 25 kDa amelogenin that is secreted into the enamel consisting of 173 amino acid residues. The 25 kDa amelogenin is seen to be lacking the region from Lys^19^ to Gln^35^ found in 27 kDa amelogenin [36]. Further studies on these fragments led to the recognition of TRAP polypeptide embedded within the N-terminal domain of native amelogenin proteins [37].

Nagano et al. [54] reported the liquid chromatography mass spectrometry LC-MS analysis of MMP-20 cleavage of native porcine amelogenin protein at site Trp^45^–Leu^46^ and at Ser^148^–Met^149^ to generate two TRAP peptides, Met^1^–Trp^45^ and Leu^46^–Ser^148^.^47^ The Met^1^–Trp^45^ (TRAP) peptide motif is further cleaved by KLK-4 at Asn^14^, Phe^15^, Lys^24^, Tyr^26^, Asn^28^, and Arg^31^ to generate smaller fragments (Figure 4d,e). MMP-20 is also seen as the primary protease responsible for generating LRAP by cleaving native porcine amelogenin N-terminal amino acid residues (Met^1^–Arg^31^) and at C-terminal amino acid residues (Met^149^–Asp^174^) facilitating the splicing resulting in the formation of LRAP peptides [53]. Further cleavage at Pro^157^ or Pro^162^ generates shorter LRAP peptides (Figure 4b,c) [55].

### 3.1. Development of Shorter Peptides from Lrap Motif

As described in Figure 4, cleavage sites generating TRAP and LRAP are seen to be conserved amongst different species. The use of shorter motifs has demonstrated the ability to initiate remineralization; therefore, murine LRAP peptides have been further investigated by Mukherjee et al. [56] to design shorter amino-acid-containing peptides of 26 and 32 residues yielding peptide P26 and P32, respectively (Table 2). The primary aims of these shortened murine amelogenin-derived peptides were to study the potential capabilities of HA binding and remineralisation initiating capabilities.

The design of P26 was driven by retaining the last 12 amino acid residues of the C-terminus and 14 amino acid residues from the N-terminus (residues Met^1^–Pro^4^, and Ser^16^ and Asn^25^), including the phosphorylation site at Ser^16^. P32 was designed in a similar manner to P26, with the addition of two polyproline repeat motifs from the midsection. To determine the initiation of HA crystal growth peptide, P26 and P32 were incubated separately in artificial saliva for 2 days. It was observed that P26 contributed in rapid crystal overgrowth (ca. ≤ 100 nm width) and was characterised as bundles of needle-like crystals, perpendicular to the enamel surface. In comparison, P32 also initiated the growth of crystals (ca. ≤ 100nm width) and was seen to be parallel to the enamel surface. The extent of HA crystal formation and size induced by P26 and P32 was compared to the full-length native murine amelogenin, which showed crystal formation of ca. ≤ 100 nm in width similar to the crystals induced by both P26 and P32 peptide. However, it was P32, which showed preferential crystal growth on the same axis as the formation of the native enamel crystals [56]. Therefore from this study, it is noteworthy to highlight the importance of P32 as a shorter peptide used for enamel remineralisation.

### 3.2. The Importance of N-Terminal Domains of Amelogenin

It was determined that the N-terminal domain embedded within the TRAP segment provides the only available phosphorylation sites (serine or threonine) within the peptide. Phosphate and calcium ions are known to aid in enamel mineralisation, therefore exploiting any functionality within the sequence that enhances phosphorylation is of interest. It is postulated that incorporation of a phosphorylation site on the peptide sequence would facilitate calcium ion binding.

**Figure 5:**
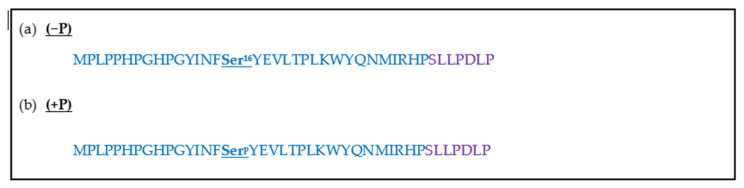
(**a**) Non-phosphorylated sequence at Ser^16^ of porcine N-terminus (blue) in the presence of partial C-terminal motif (purple). (**b**) Phosphorylated sequence at Ser^16^ of porcine N-terminus (blue) in the presence of partial C-terminal motif (purple).

Le Norcy et al. [57] studied porcine-derived amelogenin with phosphorylation (+P) and non-phosphorylation (−P) of serine at position 16 of the TRAP segment (Figure 5) [58]. The aim was to induce the formation of HA crystals by mixing the two peptide analogues in separate solutions mimicking physiological concentrations found in the tooth enamel. Peptides (−P) (Figure 5a) and (+P) (Figure 5b) were used at concentrations of 2 mg/mL in a 2.5 mM calcium and 1.5 mM phosphate containing solution at pH 7.4 and 37 °C [57]. HA crystal formation was identified by SAED, FT-IR and transmission electron microscopy TEM. It was anticipated that the peptides would act as HA crystal forming directors with the aid of the calcium and phosphate ions. A decrease in calcium and phosphate in the solution due to calcium phosphate precipitation from HA crystal formation results in a pH change, and this can be used to determine the effectiveness of each peptide in HA crystal formation.

The pH of a control sample in the absence of peptide was recorded as being ~pH 7.2. In the presence of N-terminal non-phosphorylated peptide (−P), a significant decrease in pH was observed. Mineral formation from (−P) was analysed by FT-IR, indicating that the control sample and (−P) induced similar crystal particle size. In comparison, phosphorylated peptide has a slight pH decrease, and spherical nanoparticles of calcium phosphate were observed with smaller diameters (29.1 ± 6.8 nm) when compared to calcium phosphate particles observed in the control sample (84 ± 5.2 nm).

From this study, it was deduced that the N-terminal domain alone is capable of interacting with calcium phosphate minerals to produce crystalline structures. The diameter of the different particles produced indicates the extent of the crystal formation is far more apparent with the phosphorylated peptide. The particle sizes formed by the phosphorylated peptide (~29.1 nm) are similar to particle sizes formed during natural enamel mineralisation (~20 nm) [10,58]. Therefore, the importance of the N-terminal motif contributing towards HA crystal formation is highlighted. The phosphorylation status of the N-terminus was noted for its capability to contribute to assisting mineralisation. Recognition of these domains is also validated by in vitro phosphorylation studies carried out by Nagano et al. [54].

### 3.3. Importance of the C-Terminal Domain on Formation of Particle Shape

In addition to the N-terminal domain, the presence of the C-terminal domain is recognised as having remineralisation capabilities. Some literature reports little to no influence by the native amelogenin on HA crystal nucleation and growth [59,60], whereas some report that in the absence of the C-terminal domain in the native amelogenin a significant reduction in enamel remuneration is observed [61,62]. Therefore, over the past four decades various in vitro experiments such as mineralisation experiments, HA crystal growth experiments with native amelogenin and apatite binding experiments of recombinant and native amelogenins were carried out by Beniash et al. [7], Aoba et al. [61,63], Moradian-Oldak et al. [64], respectively, to study the functionality of the C-terminal domain.

**Figure 6:**
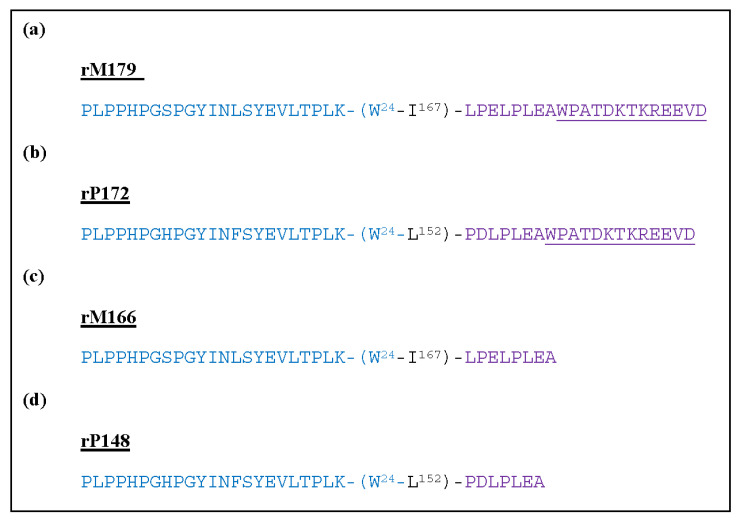
Comparison of N-terminal segment of mouse and porcine (blue) in the presence of the charged C-terminal motif (purple) with the C-terminal tail (purple and underlined) (**a**) is derived from mouse amelogenin containing the charged C-terminal tail and (**b**) porcine amelogenin containing the charged C-terminal tail. (**c**,**d**) retain the N-terminal region but without the charged portion of the C-terminal tail.

Two separate studies by Kwak et al. [58] and Wiedemann-Bidlack et al. [65] further validates the importance of the 13 amino acid residue, highly charged C-terminal tail (WPATDKTKREEVD) [22]. These studies involved the comparison of C-terminal containing recombinant amelogenin from mouse (rM179) (Figure 6a) and porcine (rP172) (Figure 6b) with C-terminal absent recombinant amelogenin mouse (rM166) (Figure 6c) and porcine (rP148) (Figure 6d). These four peptides were incubated separately for 150 min in a solution of pH 7.2 at 37 °C, closely mimicking the conditions of the oral cavity during enamel remineralisation. TEM analysis of structures formed under these conditions indicated that the peptides with the C-terminal domain of rM179 were reported as forming elongated tightly connected assemblies that are made up of chain-like structures of 7 ± 1.3nm wide and 58 ± 27.0nm long. The rP172 is reported to form structures of similar size and shape with 7 ± 2.1 nm wide and 51 ± 26.2 nm long, which are also seen to be tightly connected [65]. Peptides lacking the C-terminal domain (rM166 and rP148) were observed as forming spheres of 9 ± 2.5 nm and 20 ± 4.4nm in diameter, respectively, which are seen to be loosely connected to one another. HA crystals formed during natural mineralisation via amelogenin is reported as forming long nanorod-like structures rather than spheres [10]. The observation that the C-terminal containing peptides enable the initiation of formation of similar shapes validates the importance of this motif and highlights the key role it plays in regulating the shape and organisation of HA crystals [7].

### 3.4. C-Terminal Orientation Studies for HA Crystal Growth

Solid state NMR studies carried out by Shaw et al. [53] established that the C-terminal domain orientates itself in a manner which enhances interactions with HA crystals by orientating itself in a favourable conformation [58]. The orientation can be attributed to the charged side chains of Lys and Asp containing a direct ionic interaction with extracellular calcium and phosphate, directing HA crystal formation [58]. To study these ionic interactions, rotational echo double resonance (REDOR) NMR was employed [66]. This technique provided site specific structural information of the ionic interactions between the peptide motif and the HA crystals. The REDOR technique involves isotopic labelling of either the side chains or backbone of the C-terminal domain to measure the distance between the labelled residues to any ^31^P residues found on the surface of HA crystals. Images obtained by Le Norcy et al. [57] using TEM indicate small calcium phosphate nanoparticles that align and form needle-like structures in the presence of the C-terminal domain, which are favourable in forming nanorods and HA crystals [57]. In the absence of the C-terminal domain, flake-like structures were observed. Flake-like crystals do not have the ability to form the necessary nanoscale structures that aid HA crystal formation. The results from these experiments indicate that the C-terminal domain is orientated in a favourable conformation next to the HA crystal surface and it was concluded that the C-terminus plays an influential role in regulating the shape and organisation of HA crystal formation and growth during remineralization [67].

### 3.5. Calcium Binding Capabilities of Human LRAP (hLRAP)

The calcium-binding capabilities of human recombinant amelogenin (rH174) in comparison with the shorter LRAP version were studied by Le et al. [41] rH174 and human LRAP (hLRAP) were studied using isothermal titration calorimetry (ITC) techniques carried out in 4-(2-hydroxyethyl)-1-piperazineethanesulfonic acid (HEPES) buffer (10 mM) at pH 7.5 with a rLRAP concentration of 0.12 mM using a calcium chloride (5 mM) and a rH174 concentration of 0.064 mM with a calcium chloride concentration of 15 mM. The binding affinity of calcium to hLRAP and rH174 was determined by the number of calcium ions (N) bound to these molecules. ITC studies elucidated that hLRAP has a 6.4 higher calcium-binding affinity over the native rH174 peptide. Calcium binding is recognised as a key parameter responsible for HA crystal formation [41]. This in turn indicates the potential of employing a truncated version of full-length amelogenin as LRAP that contains both N-terminal and C-terminal motifs, can achieve the same outcome on HA crystal formation in dental mineralisation (Table 2) [68].

## 4. Successful Peptide Based Therapeutics

P11-4 is an N-terminally acetylated, C-terminal amidated 11 amino acid residue (Ac-QQRFEWEFEQQ-NH_2_) peptide It was one of five peptides (P11-1, P11-2, P11-3, P11-4 and P11-5) initially designed and synthesised as a self-assembling peptide for bone regeneration in 1997 by Aggila et al. [70] To facilitate self-assembling capabilities, the peptide sequence is arranged with alternating hydrophobic and hydrophilic amino acid residues as seen in P11-4 [71]. Studies carried out by Aggila et al. [70] established that P11-4 forms a β-sheet, which is commonly seen among self-assembling peptides. The 3D matrix formed by self-assembling P11-4 peptide is known to have high affinity towards calcium ions which acts as a nucleator facilitating de novo HA crystal formation 14]. This capability gained recognition and was applied to the regeneration of demineralised enamel. P11-4 has since then been developed into a novel peptide based therapeutic for dental caries by inducing mineralisation. It was patented and marketed as Curodont^TM^ Repair and is now widely available for the treatment of dental caries [72].

## 5. Conclusions

Structural studies carried out over the past four decades on amelogenin proteins have elucidated the importance of the N- and C-terminal domains of amelogenin on HA crystal formation and their essential role in successful in vitro enamel remineralisation to date. Phosphate and calcium ions are known to aid in enamel remineralization; therefore, exploiting the N-terminal domain by also incorporating a unique site capable of calcium binding is of great interest. In addition, the presence of the highly charged C-terminal domain is also postulated to assist in remineralisation due to its ability to adopt a favourable conformation to facilitate the initiation of remineralisation.

An area of the amelogenin midsection consisting of polyproline containing motifs has been shown to attribute to HA crystal formation while the random coil portion also present in the midsection does not greatly contribute to self-assembling properties, nor contribute in a significant manner towards HA crystal formation.

The exemplary example, Curodont^TM^ Repair, is a self-assembling peptide (P11-4), currently used by dental clinicians to induce enamel remineralisation. The physicochemical properties of the amino acid residues constituting P11-4 have shown remarkable similarities to amelogenin-derived peptides reported in the literature. Therefore, in designing future peptide-based therapeutics for enamel remineralization, the following key characteristics should be considered:

1. **Self-assembling capabilities**—To ensure that a 3D-matrix-forming scaffold is provided that will facilitate HA crystal formation and maintain similarity to natural enamel formation by the native amelogenin protein.

2. **Calcium-binding motif—**The phosphorylation of serine at position 16 in the N-terminus can assist in increasing the ability to effectively bind calcium ions, crucial to the remineralisation process.

3. **C-terminal domain motif—**Regarded as an important domain to facilitate HA formation, as the loss of the C-terminal tail led to no HA crystal formation. This highly charged tail region orientates itself in a favourable manner, allowing for ionic interactions with the HA crystal surface and thereby facilitating HA crystal formation.

4. **Polyproline motifs—**To enable HA crystal formation, eleven or more polyproline repeats are required, as having less proline repeat units has been shown to hinder HA crystal formation.

5. **Glutamine residues—**Crucial to the composition of the polyproline motifs, as they preferentially interact with proline residues of the polyproline motifs forming PPII helixes.

This brief review of the development of effective peptides that promote HA regrowth is still in its infancy. However, the key structural motifs of amelogenin protein highlighted in this review could aid in developing shorter amelogenin-derived peptides for use in enamel remineralisation.

## Figures and Tables

**Table 1:** Series of polyproline repeating motifs derived from the tail end of the mid-section (P^120^ to L^152^ represented in blue) of native human amelogenin and substitution of glutamine for alanine in PX33 to give PQA peptide. The latter most peptide resulted in unsuitable crystals for remineralisation.

Midsection of Native Human Amelogenin	YPSYGYEPMGGWLHHQIIPVLSQQHPPTHTLQPHHHIPVVPAQQPVIPQQPMMPVPGQHSMTPIQHHQPNLPPPAQQPYQPQPVQPQPHQ**P**^120^MQ**P**QP**P**VH**P**MQ^131^**P**^132^LP**P**QP**P**LP**P**MF^143^**P**^144^MQ**P**LP**P**ML^152^
**P**XX12	**P** ^120^ MQ**P**QP**P**VH**P**MQ^131^
**P**XX24	**P** ^120^ MQ**P**QP**P**VH**P**MQ^131^**P**^132^LP**P**QP**P**LP**P**MF^143^
**P**XX33	**P** ^120^ M**QPQ**P**P**VH**P**M**Q**^131^**P**^132^LP**PQ**P**P**LP**P**MF^143^**P**^144^M**QP**LP**P**ML^152^
PQA	**P**M**APA**P**P**VH**P**M**AP**LP**PA**P**P**LP**P**MF**P**M**AP**LP**P**ML

**Table 2:** Rationally designed peptides derived from native amelogenin protein to form potential mineral regeneration polypeptides.

Species	Name of Peptide	Peptide Sequence ^a^	Ref
Porcine	Porcine LRAP 56 AA	**MPLPPHPGHPGYINFSYEVLTPLKWYQNMIRHP** **SLLPDLPLEAWPATDKTKREEVD**	[57,68]
	P45	**MPLPPHPGHPGYINFSYEVLTPLKWYQNMIRHP** **SLLPDLPLEAWP**	
	P40	**MPLPPHPGHPGYINFSYEVLTPLKWYQNMIRHP** **SLLPDLP**	
Murine	Murine LRAP 59AA	**MPLPPHPGSPGYINL** **SYEVLTPLKWYQSMIRQPPLSPILPELPLEAWPATDKTKREEVD**	[56]
	P32	**MPLP-----------** **SYEVLTPLKW** **PVHPMQPS----------------** **TDKTKREEVD**	
	P26	**MPLP-----------** **SYEVLTPLKW** **PS----------------------** **TDKTKREEVD**	
Bovine	Bovine LRAP 59 AA	**MPLPPHPGHPGYINFSYEVLTPLKWYQSMIRHP** **PLPPMLPDLPLEA WPATDKTKREEVD**	[47]
	Bovine TRAP	**MPLPPHPGHPGYINFSYEVLTPLKWYQSMIRHP** **YSPYGYEPMGGT**	
Human	hLRAP 58 AA	**MPLPPHPGHPGYINFSYEVLTPLKWYQS-IRPP** **PLPPMLPDLTLEAWPSTDKTKREEVD**	[69]

^a^ Blue represents the N-terminal domain, purple represents the C-terminal domain.
